# Clusters of male and female Alzheimer’s disease patients in the Alzheimer’s Disease Neuroimaging Initiative (ADNI) database

**DOI:** 10.1007/s40708-016-0035-5

**Published:** 2016-03-30

**Authors:** Dragan Gamberger, Bernard Ženko, Alexis Mitelpunkt, Netta Shachar, Nada Lavrač

**Affiliations:** 1 Ruđer Bošković Institute, Zagreb, Croatia; 2 Jožef Stefan Institute, Ljubljana, Slovenia; 3 Tel Aviv University, Tel Aviv, Israel; 4 Jožef Stefan Institute, Ljubljana, Slovenia; 5 University of Nova Gorica, Nova Gorica, Slovenia

**Keywords:** Alzheimer’s disease, Clustering, Female and male subpopulations

## Abstract

This paper presents homogeneous clusters of patients, identified in the Alzheimer’s Disease Neuroimaging Initiative (ADNI) data population of 317 females and 342 males, described by a total of 243 biological and clinical descriptors. Clustering was performed with a novel methodology, which supports identification of patient subpopulations that are homogeneous regarding both clinical and biological descriptors. Properties of the constructed clusters clearly demonstrate the differences between female and male Alzheimer’s disease patient groups. The major difference is the existence of two male subpopulations with unexpected values of intracerebral and whole brain volumes.

## Introduction

A key issue in understanding of the Alzheimer’s disease (AD) is the recognition of relations between clinical characteristics of patients and their biological properties that can be objectively measured. Some recent studies [1] suggest the existence of different AD subtypes, and it may be expected that the identification of relevant relations is potentially easier for AD subtypes than for the complete AD population. Additionally, segmentation of the AD population may enable comparative evaluation of subpopulations of AD patients, potentially leading to a better understanding of their distinguishing properties.

An important characteristic of the proposed approach applied in this work is that clustering is performed separately for male and female populations, and that the generated patient clusters are homogeneous both in terms of clinical and biological properties. The results are relevant for identification of gender-specific properties of patients that have problems with dementia. Also, the results clearly support the conclusion that there are significant gender-related differences among AD patients [2].

Development of our clustering methodology has been motivated by the recently introduced approaches of redescription mining [3] and multi-view learning [4]. We give a detailed presentation of the proposed multi-layer clustering algorithm (MLC), since we believe that this methodology can be useful also in other medical applications. The algorithm is implemented as a web application and is easy to use. It is publicly available at http://rr.irb.hr/MLC/.

Applications of data clustering are very good examples of interactive data mining approaches [5] because evaluation of the quality of obtained clusters is only possible in the context of domain expert’s expectations [6]. Additionally, an important issue is that application of different clustering algorithms as well as different ways of data representation and preparation may result in substantially different clustering results.

Unfortunately, it was not possible to include experts participating in the ADNI into the analysis loop of our clustering of the ADNI data. Expert evaluation of the results solely with the help of publicly available data and their descriptions is difficult because some data or their aspects, especially anamnestic data and data collection procedures, are not public due to patient privacy protection issues. Nevertheless, we still used *human-in-the-loop approach* that concentrated on the identification of subpopulations that are large and interpretable with existing domain knowledge. As a result—besides some clusters that are in agreement with the existing domain knowledge—we have surprisingly identified some additional clusters that are hard to evaluate. These clusters are currently considered as potentially interesting hypotheses; their future verification on independent data might lead to new scientific insights and potentially useful medical knowledge.

The rest of the paper starts with a summary of the related work in Sect. 2 and the presentation of the data used in the analysis in Sect. 3. Section 4 describes the novel clustering methodology, including a small illustrative example. Section 5 presents the constructed clusters of ADNI patients. The clusters are described in terms of statistical properties of patients included into each cluster, together with the complete list of identification numbers of corresponding patients. In this way, the interested reader may access additional information about specific patients from the ADNI database. Medical relevance of the results, especially a possible interpretation of the unexpected clusters is discussed in Sect. 6. The quality of best biomarkers for the constructed clusters is analyzed in Sect. 7. Section 8 concludes the paper.

## Related work

The approaches suitable for the identification of relations between clinical and biological properties of AD patients can be grouped into three groups. In the first one, we have statistical approaches that typically test the significance of differences between properties of AD patients and patients from the control group. Published results obtained with these methods [7, 8] clearly demonstrate the existence of relations between the values obtained by PET imaging and the clinical diagnosis. The problem with this approach is that the identified relations are non-specific and that the severity of the disease is not strongly correlated with the measured values. The major problem seems to be that the differences in biological descriptors may be a consequence of various physiological processes and that changes of both clinical and biological variables may be a normal process in the elderly population.

Other approaches are based on data mining methods. The second group comprises supervised machine learning techniques used for identification of potentially complex relations between biological properties that strongly correlate with the AD diagnosis [9]. This is typically a very powerful approach but in the AD domain it is confronted with the problem that there exist various clinical scales of dementia but none of them can be regarded as completely reliable for determining the AD diagnosis.

The third group comprises unsupervised clustering approaches, which are very attractive because they do not require explicit definition of the target class and the availability of a control group of patients. The results often enable novel insights into the analyzed data. A good example is the identification of pathological subtypes of the Alzheimer’s disease presented in one large group characterized by the distribution of senile plaque restricted to a small number of brain regions, and a smaller group with about 15 % of patients in which the lesions were more widely distributed [10]. A general problem of clustering is instability of the results that significantly depend on the used methodology and the parameters of the algorithm selected by the user [11]. Recently, it has been demonstrated that the quality of results can be significantly improved when more than one layer of input data are used [3, 4]. The distinguishing property of the multi-layer clustering algorithm presented in this work is that—in contrast to redescription mining [3]—our algorithm does not construct descriptions of subpopulations and that—in contrast to multi-view learning [4]—it does not require statistical independence of input data layers. Additionally, the major advantage of the multi-layer clustering algorithm is that no explicit definition of the distance measure among instances (patients) is necessary and that no explicit definition of the number or size of the resulting clusters is expected from the user [12].

The first experiments with the application of the multi-layer clustering in the AD domain have been performed on 916 patients from the ADNI database described by 10 biological and 23 clinical descriptors [13]. The results demonstrated the existence of an AD subpopulation with a surprising property of the increased intracerebral and whole brain volumes. The experiments have been repeated on a set of 659 ADNI patients described with 56 biological and 187 clinical properties collected during the baseline evaluation [14]. In spite of different datasets (though some of the patients and some of the features were overlapping), again a cluster of male patients has been identified with the same surprising property of increased intracerebral and whole brain volumes. This work is an extension of these experiments on the latter dataset but with an improved clustering algorithm. The improvement resulted in identification of an additional cluster of male patients with no dementia, construction of significantly larger clusters, and elimination of obvious outliers that have been present in previous subpopulations. The basic conclusions of this work are the same as those in [14] but the statistical significance of the results is higher.

## Data

All experiments were performed on the data from the Alzheimer’s Disease Neuroimaging Initiative (ADNI) database.^1^ The total number of patients initially included was 1736 from all ADNI stages of study (i.e., ADNI-1, ADNI-GO, and ADNI-2). In order to achieve the broadest clinical dataset, some exams used only in ADNI-2 were used. Keeping only observations for which no missing values were present led to a reduction in the number of observations from 1736 to 659. This subset includes 317 female and 342 male patients. The patients are described by 147 clinical variables, 41 laboratory variables, 40 symptoms, and 15 biological measurements. Clinical variables include Alzheimer’s Disease Assessment Scale (ADAS13), Mini Mental State Examination (MMSE), Rey Auditory Verbal Learning Test (RAVLT immediate, learning, forgetting, percentage of forgetting), Functional Assessment Questionnaire (FAQ), Montreal Cognitive Assessment (MOCA), and Everyday Cognition, which are cognitive functions questionnaire filled out by patients (ECogPt) and their study partners (ECogSP) (Memory, Language, Visuospatial Abilities, Planning, Organization, Divided Attention, and the Total score), Neuropsychiatric Inventory Questionnaire, Modified Hachinski Ischemia Scale, and Geriatric Depression Scale. Examples of laboratory variables are red blood cells and total bilirubin, while examples of symptoms are palpitations and dizziness. Biological measurements include ABETA peptides, TAU and PTAU proteins, the APOE-related genetic variations (APGEN1 genotype allele 1, APGEN2 genotype allele 2), PET imaging results FDG-PET and AV45, MRI volumetric data [Ventricles, Hippocampus, Whole Brain, Entorhinal, Fusiform gyrus, Middle temporal gyrus (MidTemp) and intracerebral volume (ICV)].

For the evaluation of the consistency of constructed clusters, we use global clinical dementia rating score which is interpreted as clinically normal CN (value 0), mild cognitive impairment MCI (value 0.5) and Alzheimer’s disease AD (value 1) diagnosis for the patient. The clinical dementia rating score is different from the five level ADNI patient diagnosis (cognitive normal, significant memory concern, early mild cognitive impairment, late mild cognitive impairment, and AD), but the agreement between the two scales is very high.

In clustering, the symmetry and additivity of the variables prove to be important. Therefore, in data preprocessing, some of the variables were transformed to achieve reduced skewness. The transformation function was selected according to the type of data measured by the variable, the level of skewness, and the most adequate function from a set of possible functions, which include $\log {x}$, ${{\mathrm{logit}}}\,x$, 1/*x*, etc.

## Multi-layer clustering

In a typical machine learning setting, we have a set of examples *E* that are described by a set of attributes *A*, and from these examples we try to induce or learn a model that would generalize the examples. In some domains, the set of attributes may be partitioned in two or more disjoint subsets (layers) according to some criteria, such as the physical meaning of the attributes or the way data on specific attributes have been collected. For example, in the Alzheimer’s disease domain, the first layer can be the laboratory data, while the second layer can be the clinical data. In some other domain, different layers may contain the same attributes but collected in various time periods. The goal of multi-layer clustering is to construct clusters that are as large as possible and coherent in all the layers. This section describes the proposed clustering methodology by first describing single-layer clustering, and then generalizing it to multi-layer clustering.

### Single-layer clustering

Let us assume a basic clustering task in which we have only one layer of attributes. The proposed methodology consists of two steps. In the first step, we compute the so-called *example similarity table*. This is an * N* × * N* symmetric matrix, where *N* is the number of examples. All its values are in the range 0.0–1.0. A large value at a position (*i*, *j*), $i\ne j$, denotes a large similarity between examples *i* and *j*. In the second step, we use the table in order to construct clusters.

#### Example similarity table (EST)

We start from the original set of *N* examples described by nominal and numerical attributes that may contain unknown values. An artificial classification problem is formulated as follows: the examples from the original set constitute the positive examples, while the negative examples are artificially constructed by shuffling the values of the original examples. The shuffling is performed at the level of attributes so that we randomly mix values among the examples. The values remain within the same attribute as in the original set of examples. As a result, we have the same values in positive and negative examples, but in negative examples we have randomized connections between the attribute values. For small problems with up to 200 examples, we typically construct four times as many negative examples as in the original (positive) example set, while for larger domains we construct the same number of positive and negative examples.

Next, we use a supervised machine learning algorithm to build a predictive model that is used to discriminate between the positive examples (the original examples) and the negative examples (the artificially constructed examples with shuffled attribute values). The goal of learning is not the predictive model itself, but the information on the similarity of examples. Machine learning approaches with which we can determine if some examples are classified in the same way are appropriate for this task. For example, in decision tree learning this means that examples end in the same leaf node, while in decision rule learning this means that examples are covered by the same rule.

To estimate the similarity of examples, we follow an ensemble learning approach, where statistics are computed over a large set of classifiers. Additionally, a necessary condition for a good similarity estimation is that the classifiers are as diverse as possible and that each of the classifiers is better than random. All these conditions are satisfied, e.g., by the Random Forest [15, 16] and the Random Rules [17] algorithms. We use the latter approach in which we typically construct about 50,000 rules for each EST computation.

The similarity of examples is determined so that for each pair of examples, we count how many rules cover both examples. The EST presents the statistics for the positive examples (original set of examples). A pair of similar examples will be covered by many rules, while no rules or a very small number of rules will cover pairs that are very different in terms of their attribute values. Final EST values are computed by normalizing the counts by the largest detected value.

Table 1 presents an example of EST for a set of 6 examples. In the upper part is the table with counts of rules covering pairs of examples. The diagonal elements represent total counts of rules covering each example. By the normalization of this table, we obtain the EST that is presented in the lower part of the table. It can be noticed that we have two very similar examples (*ex2* and* ex5*), three similar examples (*ex1*,* ex3*, and* ex4*), and one very different example (*ex6*). The maximal value in the upper table is 97 and EST values in the lower table are obtained through normalization with this value.

**Table 1:** An illustrative example of a similarity table (EST)

	*ex1*	*ex2*	*ex3*	*ex4*	*ex5*	*ex6*
*ex1*	38	0	27	28	0	7
*ex2*	0	97	3	1	97	3
*ex3*	27	3	47	16	3	1
*ex4*	28	1	16	45	1	4
*ex5*	0	97	3	1	97	3
*ex6*	7	3	1	4	3	39

	*ex1*	*ex2*	*ex3*	*ex4*	*ex5*	*ex6*
*ex1*	0.39	0.0	0.28	0.29	0.0	0.07
*ex2*	0.0	1.0	0.03	0.01	1.0	0.03
*ex3*	0.28	0.03	0.48	0.16	0.03	0.01
*ex4*	0.29	0.01	0.16	0.46	0.01	0.04
*ex5*	0.0	1.0	0.03	0.01	1.0	0.03
*ex6*	0.07	0.03	0.01	0.04	0.03	0.40

#### Clustering-related variability (CRV) score

The second step in the process of clustering starts from the EST. The goal is to identify subsets of examples that can reduce the variability of EST values. For this purpose, we define the so-called *clustering-related variability* (CRV) score. This is the basic measure which guides the search during iterative bottom-up clustering. CRV score is not a simple similarity measure. It is defined for a single example, but it depends also on other examples that this example is clustered with. A cluster may consist of a single example.

Clustering-related variability, for example *i* is denoted as $\mathrm {CRV}_i$. It is the sum of squared deviations of EST values in row *i* ($X_i=\{x_{i,j},\ j\in \{1,\dots ,i-1,i+1,\dots ,N\}\}$) but so that $\mathrm {CRV}_i$ is computed as a sum of two components: $\mathrm {CRV}_i = \mathrm {CRV}_{i,{\text{wc}}} + \mathrm {CRV}_{i,{\text{oc}}}$.

Within cluster value

$$\begin{aligned} \mathrm {CRV}_{i,{\text{wc}}} = \sum _{j\in C}(x_{i,j} - x_{{\text{mean}},{\text{wc}}})^2 \end{aligned}$$

is computed as a sum over columns *j* of row *i* ($j\ne i$) corresponding to examples included in the same cluster *C* with example *i*. In this expression, $x_{{\text{mean}},{\text{wc}}}$ is the mean value of all $x_{i,j}$ in the cluster. When there is only one example in a cluster then $\mathrm {CRV}_{i,{\text{wc}}}=0$ because there are no other examples in the cluster that are different from *i*. When there are two examples in a cluster then it equals zero because we compute the sum only for one value $x_{i,j}$ and that is equal to $x_{{\text{mean}},{\text{wc}}}=x_{i,j}$.

Outside cluster value

$$\begin{aligned} \mathrm {CRV}_{i,{\text{oc}}} = \sum _{j\notin C}(x_{i,j} - x_{{\text{mean}},{\text{oc}}})^2 \end{aligned}$$

is defined in the same way as $\mathrm {CRV}_{i,{\text{wc}}}$ but for $x_{i,j}$ values of row *i* not included in cluster *C*. The $x_{{\text{mean}},{\text{oc}}}$ is the mean value of the EST element values not included in the cluster and it is different from the $x_{{\text{mean}},{\text{wc}}}$ used to compute $\mathrm {CRV}_{i,{\text{wc}}}$. When example *i* is the only example in a cluster then $\mathrm {CRV}_{i,{\text{oc}}}$ is the sum of squared deviations for all the values in row *i* except for $x_{i,i}$.

The final CRV value of cluster *C* is computed as the sum of all the CRV values for the examples contained in the cluster:

$$\begin{aligned} \mathrm {CRV}_{C}=\sum _{i\in C} \mathrm {CRV}_i. \end{aligned}$$

#### Illustrative example

We use the data from the EST presented in Table 1 to compute the CRV value for the example (*ex*1) contained in various clusters *C*. We present three cases: when cluster *C* contains only example *ex*1, when *ex*1 is clustered with *ex*3, and, finally, when it is clustered with both *ex*3 and *ex*4. By visual inspection of the EST, we can immediately notice some similarity among examples $\{ex1, ex3, ex4\}$. The goal is to demonstrate the CRV value computation to show that for the same row *ex*1, we can get different $\mathrm {CRV}_{ex1}$ values depending on which example *ex*1 is clustered with, and finally to show how $\mathrm {CRV}_{ex1}$ values decrease when similar examples are added into cluster *C*.

If *ex*1 is the only example in a cluster $C=\{ex1\}$:

$$\mathrm {CRV}_{ex1,{\text{wc}}} =0$$

$$\mathrm {CRV}_{ex1,{\text{oc}}} = (0.0-0.13)^2+ (0.28-0.13)^2+ (0.29-0.13)^2+ (0.0-0.13)^2+ (0.07-0.13)^2 =0.08$$

$$\mathrm {CRV}_{ex1} = 0.08$$

When we add a new element (ex3) to this cluster $C=\{ex1, ex3\}$:

$$\mathrm {CRV}_{ex1,{\text{wc}}} = (0.28-0.28)^2 =0.00$$

$$\mathrm {CRV}_{ex1,{\text{oc}}} = (0.0-0.09)^2+ (0.29-0.09)^2+ (0.0-0.09)^2+ (0.07-0.09)^2 =0.06$$

$$\mathrm {CRV}_{ex1} = 0.06$$

Finally, when we have $C=\{ex1, ex3, ex4\}$:

$$\mathrm {CRV}_{ex1,{\text{wc}}} = (0.28-0.285)^2 + (0.29-0.285)^2 =0.00$$

$$\mathrm {CRV}_{ex1,{\text{oc}}} = (0.0-0.02)^2+ (0.0-0.02)^2+ (0.07-0.02)^2 =0.00$$

$$\mathrm {CRV}_{ex1} = 0.00$$

#### Single-layer algorithm

Algorithm 1 is the bottom-up clustering algorithm that merges the most similar examples in respect of the CRV score, and produces a hierarchy of clusters. It may be noticed that in contrast to most other clustering algorithms, it has a well-defined stopping criterion. The process stops when further merging does not result in the reduction of example variability measured by the CRV score, and this way the algorithm automatically determines the optimal number of clusters. As a consequence, some examples may stay non-clustered (more precisely, they remain as clusters consisting of only one example).
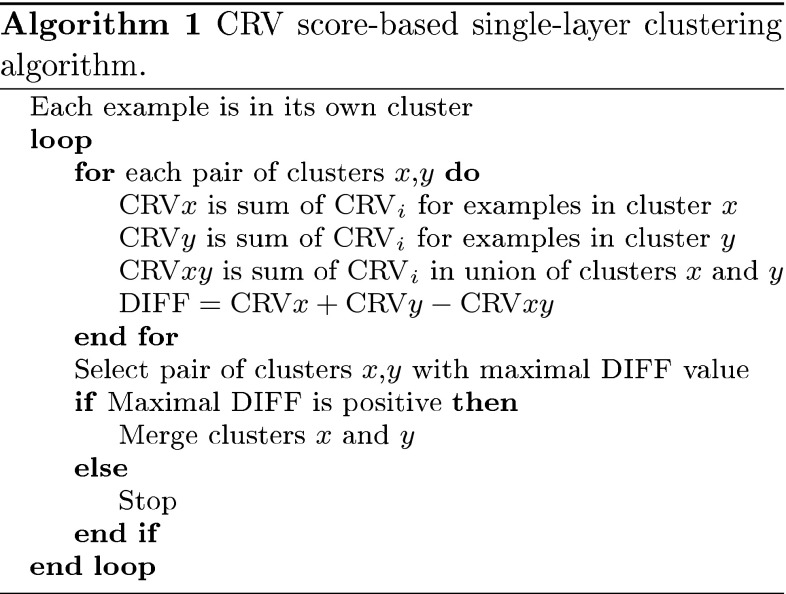


### Multi-layer clustering

The basic lesson learned from redescription mining and multi-view clustering is that the reliability of clustering can be significantly improved by a requirement that the result should be confirmed in two or more attribute layers. The approach for clustering based on example similarity, presented in the previous section for a single-layer case, can be easily extended to clustering in a multi-layer case.

If we have more than one attribute layer then for each of them we compute the example similarity table independently. For each layer, we have to construct its own artificial classification problem and execute the supervised learning process in order to determine the similarity of examples. Regardless of the number and type of attributes in different layers, the tables will be always matrices of dimension * N* × *N*. The reason is that by definition, we have the same set of *N* examples in all the layers. After computing the similarity tables, the second step of the clustering process is executed.
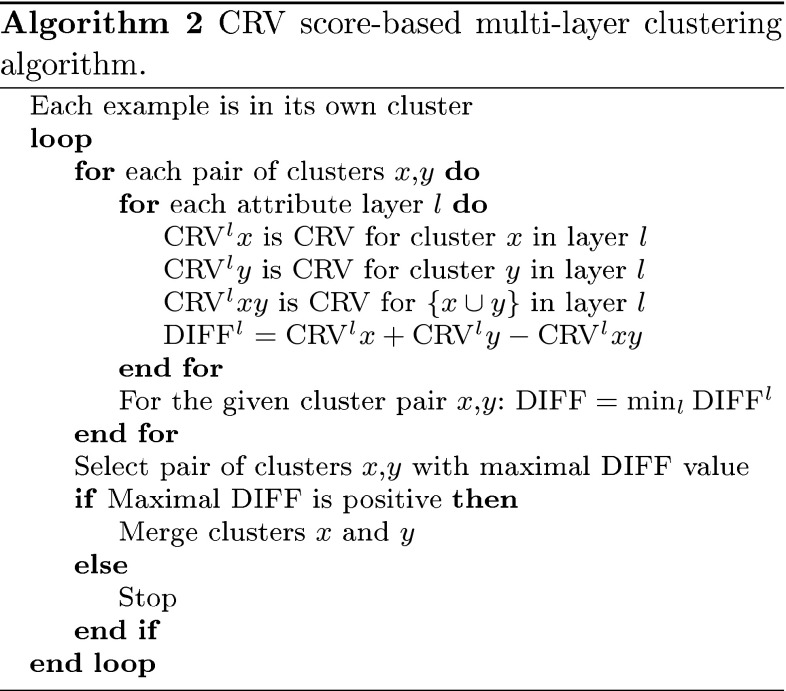


Conceptually, multi-layer clustering presented in Algorithm 2 is identical to the single-layer approach. The main difference is that merging of two clusters is possible only if there is variability reduction in all the layers. For each possible pair of clusters, we have to compute potential variability reduction for all attribute layers and to select the smallest value for this pair. If this minimal value is positive, then merging of clusters enables variability reduction in all the layers. When there are more pairs with positive minimal value, we chose the pair with the largest minimal value and merge these clusters in the current iteration.

When we do clustering in two or more layers, we have a conjunction of necessary conditions for merging two clusters. A typical consequence is that resulting clusters are smaller than in the case of a single-layer clustering.

## Clustering results

Clustering was performed independently for each of the two subpopulations of 317 female and 342 male patients. A series of experiments was performed so that different parts of available information about patients were used as input layers. The presented results were obtained by using biological measurements and laboratory data (in total 56 descriptors) as the first layer, and symptoms and clinical data (in total 187 descriptors) as the second layer.

All experiments produced a large number of clusters. For the described setting with two layers for the female population, there are 19 clusters with 4 or more patients, nine of which have more than 10 patients. The result for the male population is very similar: 21 clusters with 4 or more patients, 10 of them with more than 10 patients. Five largest clusters for each population are listed in Table 2. They include a bit more than a half of patients from each population.

**Table 2:** List of five largest clusters for female and male populations

Number of patients	Distribution of CD rating score			Cluster ID
	AD	MCI	CN	
Females				
64	0	5	59	F0
47	19	27	1	F1
22	0	20	2	–
20	0	0	20	–
19	3	15	1	–
Males				
42	0	12	30	M0A
40	0	8	32	M0B
38	18	20	0	M1
31	13	18	0	M2
27	0	26	1	–

Table 3 presents the clinical and biological properties for six clusters that include more than 30 patients. For the female population, we have one large cluster F1 in which the majority of patients have significant problems with dementia. Out of the 47 included patients, 19 have the Clinical Dementia rating score equal to 1 (in this work interpreted as AD), while 28 have been diagnosed as mild cognitive impairment (CD score of 0.5). In the entire dataset, there are 22 patients with the score value equal to 1, and 19 of them are included into this cluster. The clinical properties of these patients include high ADAS13, FAQ, and MMSE scores, and all types of cognitive problems. The biological properties of these patients are also typical for AD patients, e.g., low FDG values, significantly decreased Entorhinal volume, and high AV45 values. A statistical comparison with the population of all 145 female patients with cognitive normal status in the dataset has been used to identify the most distinguishing biological properties of the cluster. The last column of Table 3 presents the most significant properties in terms of the highest z-score values of the Mann-Whitney test. The values are very high denoting that differences between cognitive normal patients and those included in the cluster are very significant.^2^

**Table 3:** Short descriptions for the largest clusters

Cluster ID	Clinical status	Biological properties (with * z* score versus cognitive normal)	
Clusters for female patients			
F1	Significant cognitive problems (high ADAS13, high FAQ, high MMSE)	Low FDG	9.29
		Low entorhinal	8.74
		High AV45	8.36
		Low hippocampus	8.33
		Low MidTemp	7.52
		Low fusiform	7.13
		High TAU	7.12
F0	Mild or no dementia	High FDG	6.05
		High hippocampus	2.86
		High whole brain	2.85
Clusters for male patients			
M1	Significant cognitive problems	Low FDG	8.23
		Low hippocampus	7.70
		Low entorhinal	7.09
		Low MidTemp	6.33
		Low whole brain	6.23
		High TAU	5.57
M2	Significant cognitive problems	Low FDG	7.83
		High ICV	6.54
		High ventricles	5.70
		Low hippocampus	5.60
		Low ABETA	5.80
M0A	Mild or no dementia	High FDG	4.98
		High ICV	4.00
		High whole brain	3.31
M0B	Mild or no dementia	Low ICV	4.83
		Low whole brain	3.74

Cluster F0 constructed for the female population includes 64 patients that are typical patients with no significant problems with dementia. Although this is the largest cluster constructed for the female population, it is relatively small if we take into account that there are 145 cognitive normal female patients in the whole dataset. A possible explanation is that among ADNI patients diagnosed as cognitive normal there are also patients that are not completely healthy, but their subjective or objective problems are either not severe enough or their problems are in discrepancy with typical clinical profiles.

The bottom part of Table 3 presents clusters for the male population. There are two clusters of patients with significant cognitive problems (M1 and M2) and two clusters of patients with mild or no dementia (M0A and M0B). Cluster M0A is similar in terms of the properties of the female cluster F0 both regarding its size and the detected biological properties. It is interesting to notice that cluster M0A includes even 12 patients that have CD rating score equal to 0.5 while in the female cluster there are only five such patients. In contrast to cluster M0A, cluster M0B is characterized by decreased ICV values.

There are two clusters of male patients that have significant problems with dementia. In the first one (M1) there are 38 patients, 18 of them with the CD score equal to 1 and the rest with the score equal to 0.5. In the second cluster (M2) there are 31 patients, 13 of them with CD score equal to 1. In the male population, there are a total of 36 patients with AD status (CD value equal to 1). An interesting observation is that two male clusters M1 and M2 together include 31 out of 36 (86 %) male patients with CD score equal to 1 in the dataset, while the single female cluster F1 includes almost identical percentage of such patients (19 out of 22, i.e., 86 %). Table 4 lists the ADNI IDs of patients included into clusters F0, F1, M0A, M0B, M1, and M2.

**Table 4:** Lists of ADNI patients included into clusters from Table 3

Cluster F1													
**4024**	4030	4034	4058	4079	**4201**	4209	**4211**	**4252**	4324	**4353**	4402	4415	4458
**4477**	**4500**	4502	4542	4568	**4591**	4609	4660	4715	4796	4815	4845	4894	4897
4902	4904	**4905**	**4906**	4909	**4910**	4912	4918	**4982**	**4984**	**4990**	**4997**	**5006**	**5015**
**5019**	5031	5063	**5119**	5184									
Cluster M1													
**4009**	**4095**	4096	4131	**4152**	4171	**4195**	4215	4240	4307	4475	**4494**	4501	4526
4625	4672	4686	4689	**4707**	**4718**	**4770**	4774	**4802**	4827	4857	**4867**	4936	4958
**4964**	**4968**	4980	4994	**5017**	5027	**5067**	**5165**	**5224**	**5241**				
Cluster M2													
**4136**	4153	4192	**4223**	4243	4258	4346	4423	4515	4546	4549	4595	**4615**	4661
**4692**	**4733**	**4859**	**4863**	**4924**	4943	**4971**	4974	5012	**5037**	5058	**5059**	5070	**5071**
5095	**5208**	5210											
Cluster F0													
4028	4066	4076	4084	4155	**4184**	4200	4288	4320	4335	4340	4349	4357	4362
4399	4401	4422	4441	4446	4483	4496	4508	4545	**4553**	4555	4598	4607	4624
4643	4644	4645	4843	4872	**4874**	4878	4900	4952	5093	5102	5118	5127	5129
5132	5154	5158	5159	5169	5175	5185	5193	5198	5203	5214	5230	5235	**5240**
5261	5272	5277	5287	5288	5289	5290	5292						
Cluster M0A													
**4029**	4037	4043	4082	4164	4177	4179	**4210**	4225	**4229**	4257	**4274**	**4309**	**4332**
4339	4345	4352	4389	4427	4429	4431	4453	4485	4516	4520	**4556**	4604	4632
4649	4739	**4844**	4921	**4926**	**4941**	**4966**	5113	5131	5141	5157	5242	5271	5296
Cluster M0B													
4086	4090	4103	4158	**4168**	4176	**4251**	4292	4369	4391	4400	**4443**	4464	4469
4491	4577	4579	**4601**	4620	4762	**4799**	**4813**	4862	**4877**	5082	5083	5109	5130
**5135**	5147	5150	5167	5212	5243	5248	5250	5266	5278	5279	5294		

For clusters F1, M1, and M2, RIDs of patients with the diagnosis of the Alzheimer’s disease (CDGLOBAL value equal to 1) are typeset in bold, while for clusters F0, M0A, and M0B, patients with the MCI diagnosis (CDGLOBAL value equal to 0.5) are typeset in bold

## Analysis of results

A significant difference between male and female populations of patients can be noticed. For the female population there are two clusters while for the male population there are four clusters, two for patients with significant problems with dementia and two for patients with mild or no dementia. By inspecting the properties characterizing patients in these clusters (see Table 3), one can notice especially interesting differences between patients in clusters M1 and M2. Biological and clinical properties that most significantly differentiate these two clusters according to the Mann-Whitney test are listed in Table 5.

**Table 5:** Biological and clinical properties that are most significantly different for patients in clusters M1 and M2

Property	Average value for cognitive normal males	Average value for M2	Average value for M1	Mann-Whitney * z* score M1 versus M2
Biological properties				
ICV (*1000)	**1577**	**1774**	**1479**	6.98
Whole brain (*1000)	**1109**	**1167**	**983**	6.12
MidTemp	21629	20127	17930	3.84
Hippocampus	7808	6530	5722	3.47
Fusiform	19593	18457	16672	3.37
Ventricles	35686	64375	47414	2.47
Clinical properties				
Abstraction_moca	1.81	1.64	1.14	2.85
Neuropsychiatric Inv. (impatience)	0.29	0.52	1.78	2.07
Naming_moca	2.92	2.82	2.38	1.78
FAQTV	0.10	1.97	2.62	1.62

Cluster M2 deserves special attention due to the fact that average values of ICV and whole brain volume for patients in M2 are *higher* than average values for the set of all 124 cognitive normal male patients. The result is unexpected because cognitive problems are typically related with the atrophy of human brain [18]. The differences are statistically significant; average ICV values are 1,577 and 1,774 for cognitive normal and M2 patients, respectively (* z* score 6.54, * P* < 0.001) (see Table 3), while average whole brain volumes are 1109 and 1167 (* z* score 3.08, * P* < 0.01).^3^ When comparing patients in cluster M2 with patients in M1, who also have typical AD symptoms but, as expected, decreased ICV and whole brain volumes, the differences are even more statistically significant (see Table 5).

The importance of the discovery is manifold. First, it indicates gender-specific differences because such a cluster with similar properties is not detected in the female population. Second, for a domain in which biological processes with opposite manifestations (decrease and increase of ICV) may result in similar clinical consequences (dementia), segmentation of the patient population is suggested before other analyses aimed at the discovery of relations between biological and clinical properties of patients are performed. Finally, the result is intriguing in respect of its biological and medical interpretation.

It is possible that the increased ICV and whole brain volumes are a consequence of an artifact in data collection procedures, feature extraction from images, or data post-processing (normalization). The assumption may stimulate careful evaluation of the ADNI data, especially for patients in cluster M2. But the result may also suggest the existence of a different biological pathway for the male population, resulting in serious dementia problems that are often diagnosed as Alzheimer’s disease but with less expressed clinical symptoms (see bottom part of Table 5). In the scientific literature, we have found no support for such explanation except that the study devoted to gender-related differences [2] concluded that “AD pathology is more likely to be clinically expressed as dementia in women than in men.”

**Fig. 1:**
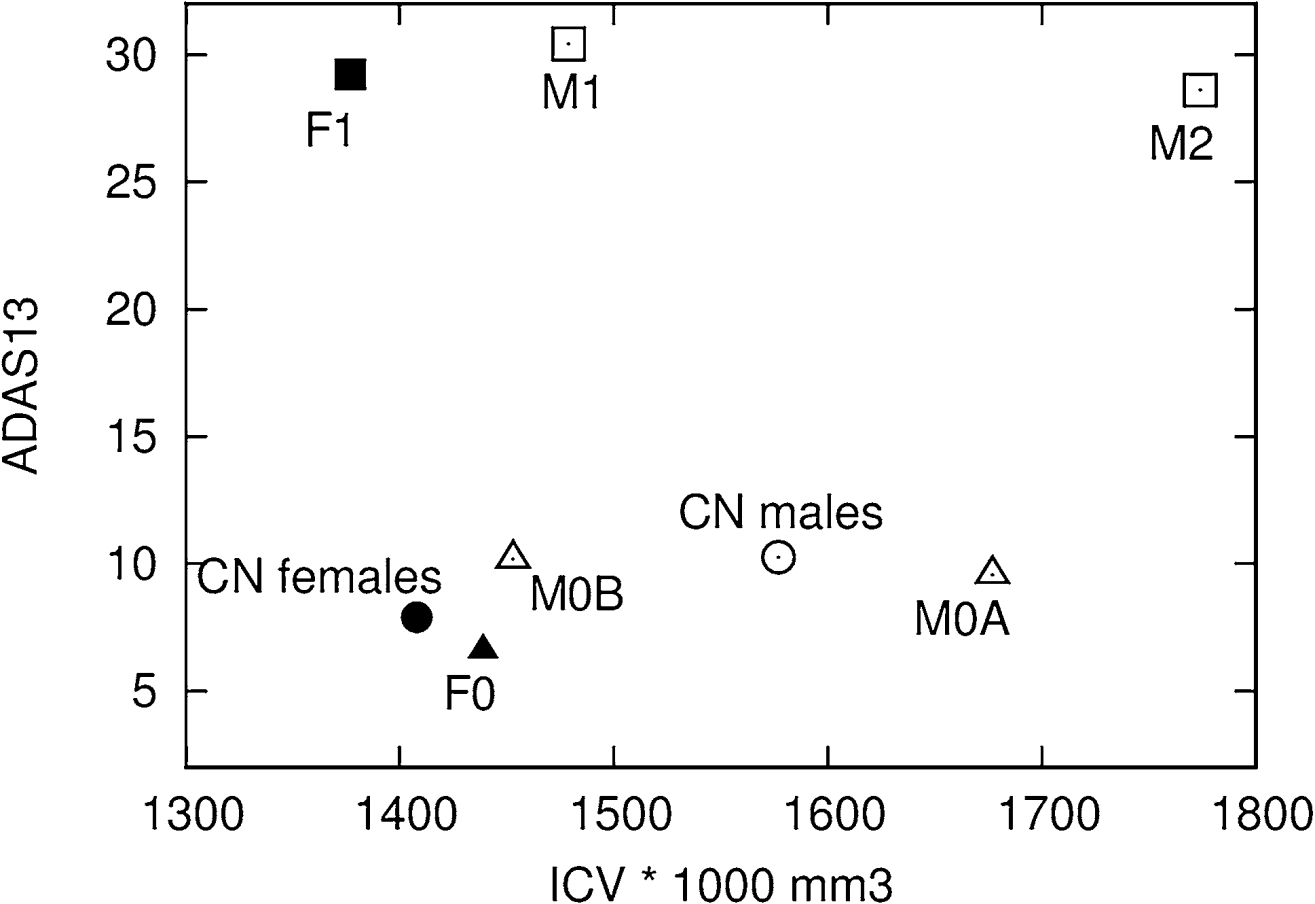
Average values of Alzheimer’s Disease Assessment Scale (ADAS13) and intracerebral volume (ICV) for all females with clinical dementia rating score equal zero (*black circle*), females in cluster F0 (*black triangle*), females in cluster F1 (*black square*), all males with clinical dementia rating score equal zero (*white circle*), males in clusters M0A and M0B (*white triangles*), and males in clusters M1 and M2 (*white squares*)

Figure 1 illustrates the differences among patients in clusters M1 and M2 and cognitive normal male patients in respect of ICV values and ADAS13 scores. It can be noticed that male cluster M0A can be compared with female cluster F0 because they share common properties: increased ICV and decreased ADAS13 score when compared to mean values of all cognitive normal male and female patients, respectively. Cluster M0B is again a surprise because it represents a group of patients which also has improved (lower) ADAS13 values but with *decreased* values of ICV. The differences between cluster M0B and the complete cognitive normal male patients are not statistically significant but the result additionally stresses differences between male and female populations and suggests that the differences between clusters M1 and M2 that are valid for AD patients are to some extent present also in the cognitive normal population.

## Biological markers

One of the stated ADNI goals is to improve clinical trial design through detection of biomarkers that could be used as approximate measures of the severity of dementia. This is known as a difficult task that is still far from a satisfactory solution. If the constructed clusters are really more homogeneous than the complete population, then it may be expected that identification of dementia disease markers should be an easier task for each cluster separately than it is for the complete population.

**Table 6:** Most correlated biological–clinical pairs of properties for various patient populations

Population	Number of patients	Biological property	Clinical property
	Spearman correlation * r*_*s*_		
All	659	FDG	MOCA
	corr. –0.51	(df = 645, * P* < 0.001)	
Female	317	FDG	ADAS13
	corr. –0.56	(df = 307, * P* < 0.001)	
Male	342	Hippocampus	ADAS13
	corr. –0.58	(df = 289, * P* < 0.001)1	
F1	47	MidTemp	ADAS13
	corr. –0.53	(df = 45, * P* < 0.001)	
M1	38	Basophils	FAQ
	corr. –0.71	(df = 32, * P* < 0.001)	
M2	31	APGEN2	RAVLT.forg.
	corr. 0.70	(df = 23, * P* < 0.01)	

Table 6 presents the most correlated pairs of one biological and one clinical property that can be identified for the complete population, for the female population only, for the male population only, and finally for clusters F1, M1, and M2. The most correlated pairs are identified with the Spearman rank-order correlation coefficient $r_s$ that is computed for all possible pairs of properties. The result confirms that for some constructed clusters there exist more strongly correlated biological–clinical relations.^4^ It must be noted that in spite of a high correlation coefficient value, the statistical significance of correlation for the smallest cluster is smaller than for the larger clusters because of its size. The result means that detected high correlation is not so reliable and that it has to be confirmed by further experiments. As expected, FDG is the most useful biological property for the general patient population and the result is in agreement with previously reported research [7].

## Conclusions

The presented results confirm that novel machine learning approaches to clustering can indeed be a useful tool for identifying homogeneous patient subsets in various medical knowledge discovery tasks. The applied multi-layer clustering technique and its combination with the gender-related separation of the population of patients is definitely not the only possible approach but its results are promising. Still, significant further research effort in this direction is necessary. Clusters constructed with the multi-layer clustering are small and six largest clusters together contain only about 40 % of all patients. In spite of this, the analysis of the results supports the conclusion that there are significant gender-specific differences in Alzheimer’s disease. Additionally, for the male population, two subpopulations with surprising properties have been detected: A subpopulation of AD patients with *increased* ICV and whole brain volumes and a subpopulation of cognitive normal patients with *decreased* ICV volume. The result suggests that segmentation of the AD patient population is strongly recommended as a preprocessing step for any analysis aimed at understanding of relations between biological and clinical properties of AD patients; however, based on the available data, we still do not know how to practically perform the segmentation in a non ad-hoc manner for the majority of patients with cognitive problems.

In future work, we plan to compare multi-layer clustering with redescription mining and to test if results of redescription mining might be used for human understandable interpretation of clusters obtained by the multi-layer approach. Regarding medical evaluation, we plan to test if it is possible to identify M1 and M2 clusters on non-ADNI patients. The ultimate goal would be to better understand differences between these two populations.

## Footnotes

1 The ADNI was launched in 2003 by the National Institute on Aging (NIA), the National Institute of Biomedical Imaging and Bioengineering (NIBIB), the Food and Drug Administration (FDA), private pharmaceutical companies and non-profit organizations. The Principal Investigator of this initiative is Michael W. Weiner, MD, VA Medical Center and University of California, San Francisco. More information can be found at http://www.adni-info.org and http://adni.loni.usc.edu.

2 A value of a * z* score higher than 3.29 denotes statistical significance of * P* < 0.001.

3 Actual absolute values for ICV and whole brain volumes are 1000 times larger.

4 Only the absolute value is important, the negative sign means an inverse correlation.
